# Optical Performance of Top-Down Fabricated AlGaN Nanorod Arrays with Multi-Quantum Wells Embedded

**DOI:** 10.1186/s11671-019-3003-1

**Published:** 2019-05-21

**Authors:** Shucheng Ge, Jiangping Dai, Na Gao, Shiqiang Lu, Penggang Li, Kai Huang, Bin Liu, Junyong Kang, Rong Zhang, Youdou Zheng

**Affiliations:** 1 Department of Physics, Fujian Key Laboratory of Semiconductor Materials and Applications, Collaborative Innovation Center for Optoelectronic Semiconductors and Efficient Devices, Xiamen University, Xiamen, 361005 China; 2 Jiangsu Provincial Key Laboratory of Advanced Photonic and Electronic Materials, School of Electronic Science and Engineering, Nanjing University, Nanjing, 210093 China

**Keywords:** DUV AlGaN multi-quantum wells, Nanorod light-emitting diodes, Internal quantum efficiency, Light extraction

## Abstract

Deep ultraviolet AlGaN-based nanorod (NR) arrays were fabricated by nanoimprint lithography and top-down dry etching techniques from a fully structural LED wafer. Highly ordered periodic structural properties and morphology were confirmed by scanning electron microscopy and transmission electron microscopy. Compared with planar samples, cathodoluminescence measurement revealed that NR samples showed 1.92-fold light extraction efficiency (LEE) enhancement and a 12.2-fold internal quantum efficiency (IQE) enhancement for the emission from multi-quantum wells at approximately 277 nm. The LEE enhancement can be attributed to the well-fabricated nanostructured interface between the air and the epilayers. Moreover, the reduced quantum-confined stark effect accounted for the great enhancement in IQE.

## Introduction

In the past decade, AlGaN-based UV LEDs have attracted wide attention because of their promising applications such as water purification, sterilization, and biochemical detection. [1–3]. Compared with traditional mercury UV lamps, AlGaN-based UV LEDs are robust, compact, and environmentally friendly and can be turned on without warming up step. However, strong piezoelectric field exists in the AlGaN multi-quantum wells (MQWs), resulting in spatial separation of electrons and holes, named as quantum-confined stark effect (QCSE), which dramatically decreases the internal quantum efficiency (IQE) [4]. Another problem is the low light extraction efficiency (LEE) [5], which is caused not only by the internal total reflection at the epilayers’ interface, but also by the dominant transverse magnetic (TM) polarized light [6]. Previous investigations suggested that the energy band engineering is an effective way to reduce the QCSE and thus improve the IQE [7]. On the other hand, the interface engineering, such as incorporating structures like photonic crystal [8, 9], patterned substrate [10, 11], distributed Bragg Reflector [12], and surface plasmons [13–16], can enhance the LEE of the deep UV LEDs. However, the combination of these methods is relatively difficult.

Fabrication of AlGaN-based deep UV nanostructured LEDs can be an alternative way to overcome QCSE and low LEE issues at the same time. Generally, nanostructured LEDs were fabricated by nanometers scale masks and top-down dry etching techniques. The masks were prepared via annealed metal nanoparticles such as nickel (Ni) or gold [17, 18], nanosphere lithography [19–21], electron beam lithography (EBL) [22], and focused ion beam milling [23]. Meanwhile, several selective area epitaxy methods have been developed to obtain InGaN-based nanowire LEDs [24, 25]. However, each method has its own natural disadvantages, such as expensive, uncontrollable morphology, non-uniform, incompatible with microelectronics processes, and time-consuming. In order to overcome these shortcomings, we have developed a soft UV-curing nanoimprint lithography (NIL) technique to prepare controllable masks in a very large area, with high uniformity and low density of defects [26, 27].

In this work, we successfully prepared AlGaN nanorod (NR) arrays with MQWs embedded from planar AlGaN LED wafers. Compared with the planar (PLA) samples, 1.92-fold LEE enhancement and 12.2-fold relative IQE enhancement have been demonstrated. Cathodoluminescence (CL), scanning electron microscopy (SEM), and transmission electron microscopy (TEM) measurements suggested that the enhanced LEE can be attributed to the improved interfacial quality between the air and the epilayers. The Raman measurements demonstrated that the strain in the MQWs is reduced from 0.42% to 0.13%, which is beneficial for IQE enhancement.

## Methods

The AlGaN LED wafer was grown by metal-organic chemical vapor deposition (MOCVD) on a 2 in. *c* plane sapphire substrate, which is defined as the PLA sample. The epitaxy comprised a 900-nm undoped AlN buffer, a 400-nm graded Al composition AlGaN layer, a 1.4-μm-thick Si-doped n-Al_0.5_Ga_0.5_N, and 5 periods of Al_0.35_Ga_0.65_N/Al_0.45_Ga_0.55_N MQWs with well and barrier thickness of 3 and 10 nm, respectively, followed by a 100-nm Mg-doped p-GaN contact layer.

**Fig. 1:**
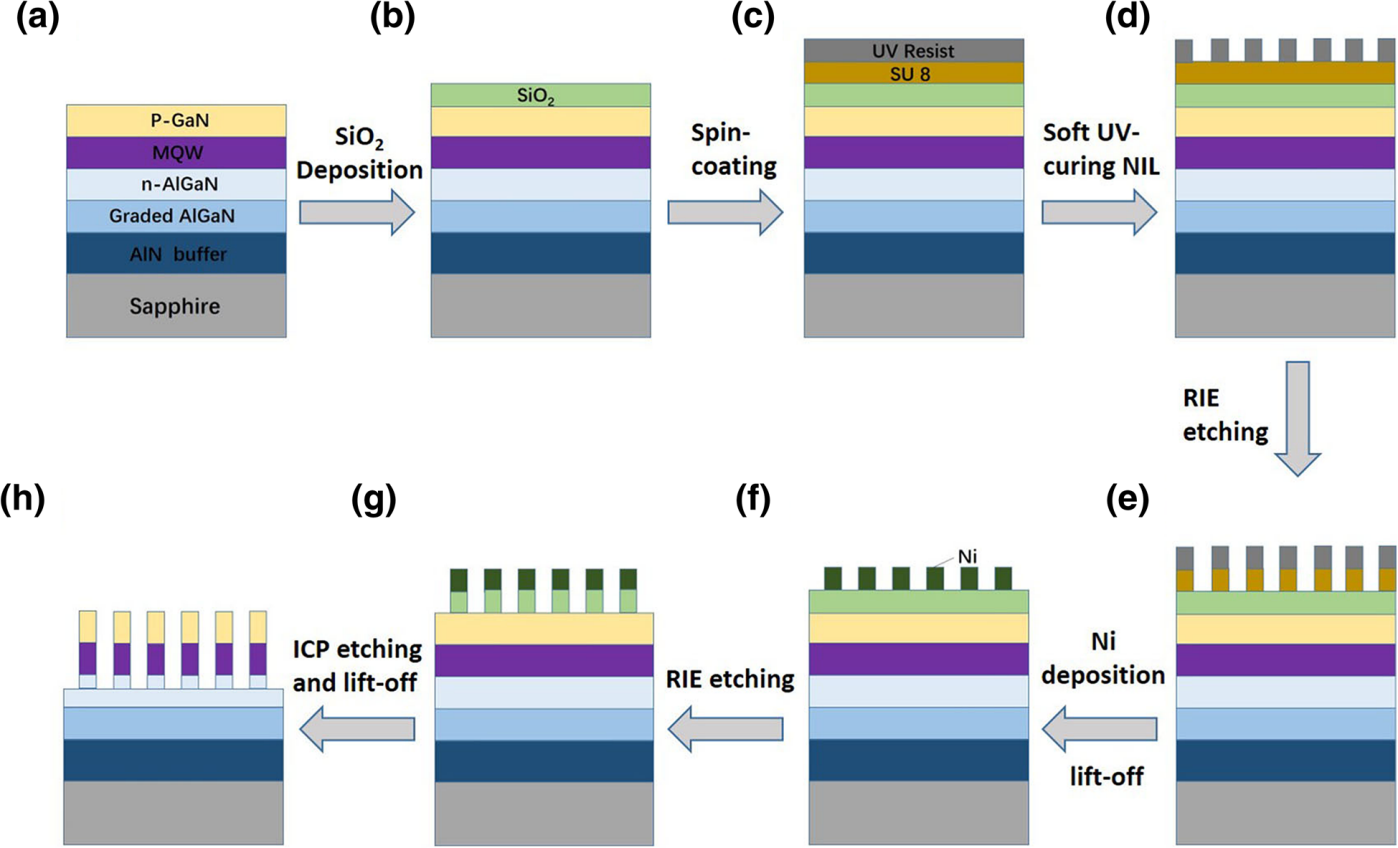
Schematic of the fabrication of AlGaN NR arrays with MQWs embedded. **a** Original AlGaN-based deep UV LED wafer. **b** SiO_2_ deposition. **c** Spin-coating process of the SU8 photoresist and the UV curable resist. **d** Soft UV-curing NIL process. **e** RIE process of SU8 photoresist. **f** Ni deposition and lift-off process in acetone. **g** Transfer Ni patterns to the SiO_2_ layer by RIE. **h** Transfer patterns from SiO_2_ to AlGaN-based LED wafer by ICP

A soft UV-curing NIL and a post-growth etching approach have been employed to obtain the AlGaN NR arrays [26–28]. As shown in Fig. 1a–h, NIL process started with a deposition of a 200-nm-thick silicon dioxide (SiO_2_) by using plasma-enhanced chemical vapor deposition (PECVD) method (Fig. 1b). Then, a layer of 300-nm-thick SU8 photoresist and a layer of 80-nm-thick UV curable resist were directly spin-coated on the epilayer (Fig. 1c), with post soft UV-curing NIL on the UV curable resist (Fig. 1d). To remove the UV resist residue and duplicate the nano-patterns to the SU8 photoresist layer, oxygen (O_2_) plasma was utilized to etch SU8 photoresist via reactive-ion etching (RIE) procedure (Fig. 1e). After that, a 30-nm-thick Ni layer was deposited via physical vapor deposition (PVD), and lift-off process followed to form periodic Ni islands on the surface of SiO_2_ layer, which served as the hard mask (Fig. 1f). The prepared Ni hard mask was used to transfer the patterns to SiO_2_ layer by another RIE process (Fig. 1g). Subsequently, these SiO_2_ nanorod arrays were employed as a second mask to etch the AlGaN LED wafer via an inductively coupled plasma (ICP) etching process. Finally, these SiO_2_ nanorod array masks were removed by HF solution, and AlGaN NR arrays were acquired as depicted in Fig. 1h. The yield of nanostructures with this NIL technology is over 98% on 2 in. wafer, which is comparable with the EBL method but the NIL technology is much cheaper. The details could be found in our previous report [27]. It is inevitable to generate surface states on the sidewall of the nanorods during dry etching, which may serve as non-radiation recombination centers and suppress the luminescence of AlGaN MQWs. Thus, all the NR samples have undergone a chemical treatment by using KOH and dilute acid solution at 90 °C in water bath to remove the surface states.

The morphology of fabricated AlGaN NR arrays was characterized in a ZEISS SIGMA high-resolution field emission SEM system. TEM images were collected by FEI Titan 80-300 TEM system with electron beam operating at 200 kV. The CL spectra were collected by an electron beam-fiber probe system with electron beam operating at 10 kV and 922 pA. The spectra of Raman scattering was obtained in a Confocal Raman spectroscopy imaging system (WITec alpha 300RA) with backscattering configuration, by using a 514-nm laser as excitation source. The Raman measurement was calibrated by a standard single crystal silicon sample with optical phonon mode at 520.7 cm^−1^.

## Results and Discussion

**Fig. 2:**
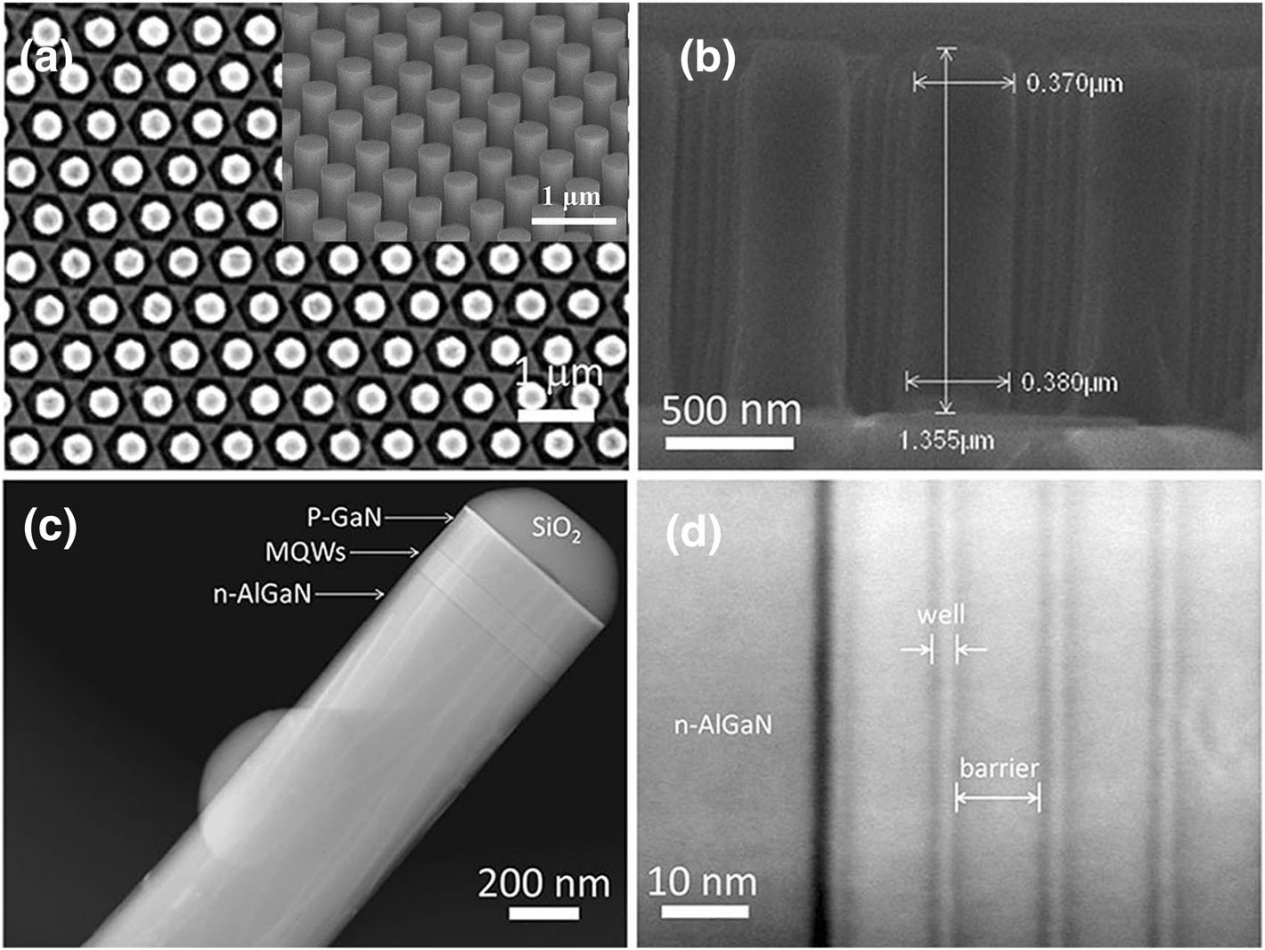
The top view (**a**), titled (inset in a), and cross-sectional (**b**) SEM images of the AlGaN NR arrays. **c**, **d** the TEM images of single NR and AlGaN MQWs, respectively

Figure 2 a, inset in a, and b show the typical top view, titled, and cross-sectional SEM images of the fabricated AlGaN NR arrays with good uniformity and smooth sidewalls. One can see that the NR are in a highly ordered hexagonal array. The diameter, period, and length of the NRs are approximately 350 nm, 730 nm, and 1300 nm, respectively. As shown in Fig. 2 c and d, the MQWs embedded in the NR can be clearly observed after NR fabrication. The well and barrier are presented as dark and bright areas, respectively, and the interface is still legible, flat, and steep.

**Fig. 3:**
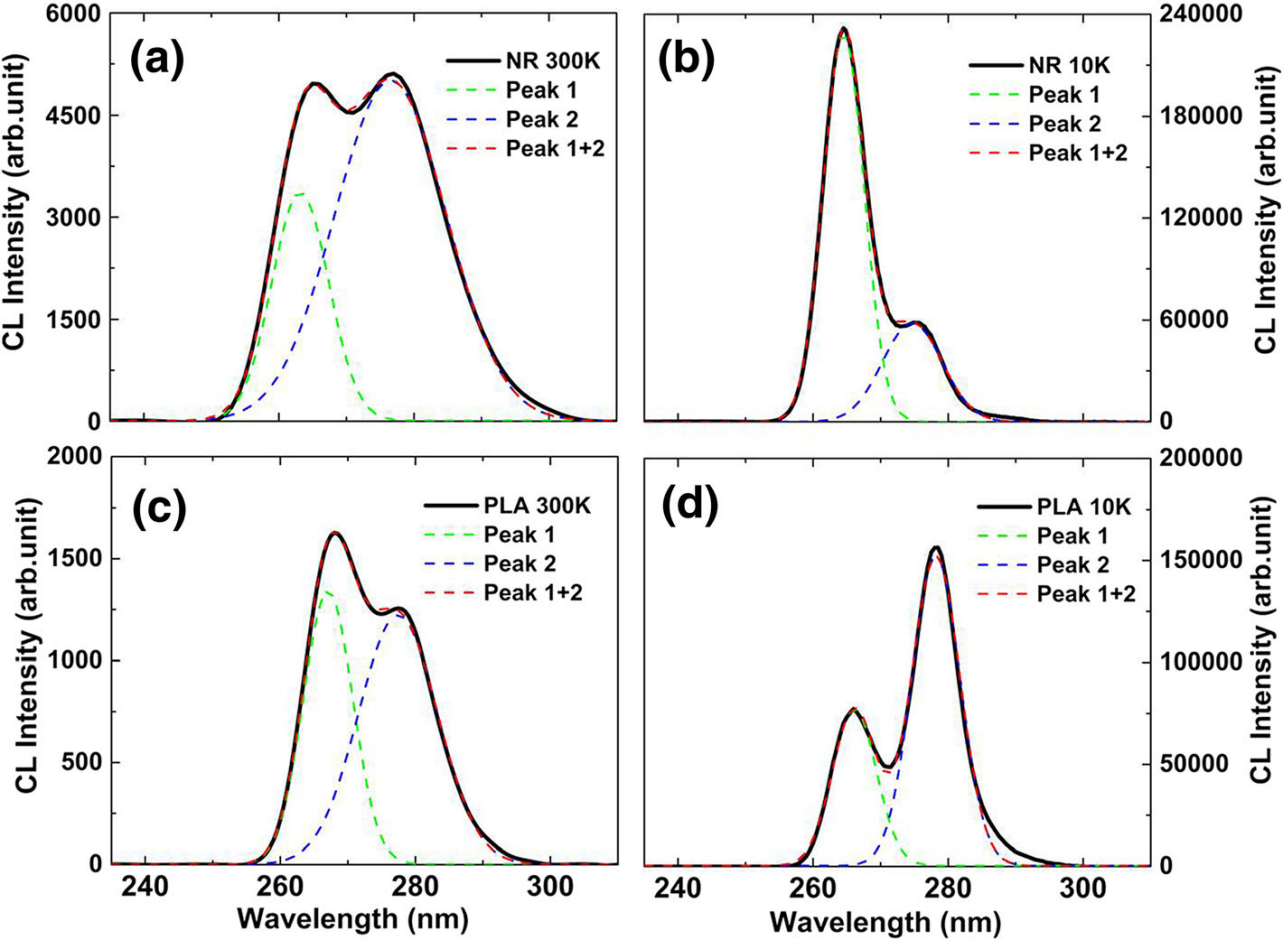
**a**, **b** CL spectra of NR samples at 300 K and 10 K, respectively, excited by an electron beam (10 kV, 992 pA). **c**, **d** CL spectra of PLA samples at 300 and 10 K, respectively, excited by an electron beam (10 kV, 992 pA). The solid lines and dash lines are corresponding to the experimental and Gaussian fitting curve

Figure 3 a and b show the room temperature (RT; 300 K) and low temperature (LT; 10 K) CL spectra of NR samples, respectively. Figure 3 c and d show the RT and LT CL spectra of the PLA samples, respectively. The solid lines and dash lines are experimental and fitted curve (Gaussian). Gaussian fitting indicates that all spectra consist of two emission peaks. Regardless of the PLA or NR sample, the CL luminescence intensities measured at LT exhibit a great enhancement compared with those under RT. This can be attributed to the weak thermal activation energy at LT. Thus, the carriers cannot migrate to defects where carriers can be non-radiatively recombined, which means that the carriers only perform radiation recombination and the IQE can be regarded as approximately 100%. Considering the structure of the epitaxial layer, the peaks at short (Peak 1) and long (Peak 2) wavelengths are attributed to the emissions of n-type layer and MQW, respectively. The detailed parameters obtained from the Gaussian divided peaks are shown in Table 1. For the NR sample, the integrated intensities of emission from the n-type layer are approximately 2.89 [*I*_1_(NR300K)/*I*_1_(PLA300K)] and 2.78 [*I*_1_(NR10K)/*I*_1_(PLA10K)] times higher than that for the PLA sample at RT and LT, respectively. However, at RT, the integrated intensity of the emission from the MQW for NR sample is approximately 5.81 [*I*_2_(NR300K)/*I*_2_(PLA300K)] times higher than that of PLA sample, while the ratio is only 0.48 [*I*_2_(NR10K)/*I*_2_(PLA10K)] at LT.

**Table 1:** The integrated peak intensities of the NR and PLA samples at 300 K and 10 K

	Peak 1 integrated intensity *I*_1_(a.u.)	Peak 2 integrated intensity *I*_2_(a.u.)
NR 300 K	3.54 × 10^4^	1.04 × 10^5^
NR 10 K	1.74 × 10^6^	6.46 × 10^5^
PLA 300 K	1.23 × 10^4^	1.79 × 10^4^
PLA 10 K	6.27 × 10^5^	1.36 × 10^6^

**Fig. 4:**
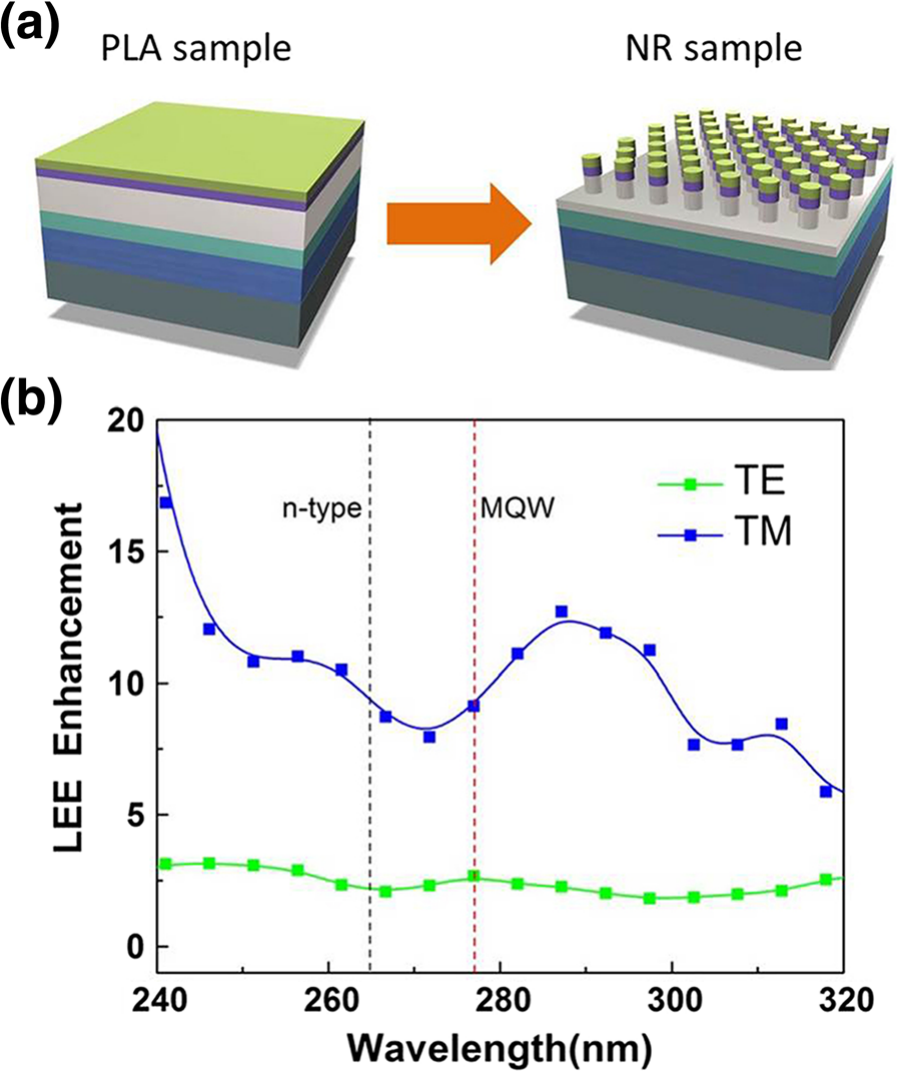
**a** The structure diagram of PLA and NR sample. **b** The LEE enhancement of NR sample compared with PLA sample at TE and TM polarization state calculated by FDTD simulation. The black and red dashed lines correspond to the emission wavelength of n-type AlGaN layer and AlGaN MQWs, respectively

Compared with PLA sample, the sidewalls of the NR sample are exposed to the air as shown in Fig. 4a, resulting in a significant increase of the total interface area between the air and the epilayer. Thus, the LEE can be enhanced for both n-type layer and MQW emissions. The LEE enhancement of the n-type layer emission can be estimated to be approximately 2.8 [*I*_1_(NR)/*I*_1_(PLA)]. Moreover, according to the geometric structure obtained from Fig. 2a, the MQW area of the PLA sample is approximately 4 times larger than that of the NR samples. By assuming the IQEs for both PLA and NR samples as 1 at 10 K, the relative light extraction enhancement can be obtained as approximately 1.9 [4 × *I*_2_(NR10K)/*I*_2_(PLA10K)]. Clearly, the LEE enhancement of the n-type layer emission is higher than that of the MQW emission.

Finite-difference time-domain (FDTD) simulations were performed to clarify the LEE enhancement of AlGaN NR arrays. The diameter, period, and length of the NR arrays are set as 350 nm, 730 nm, and 1300 nm, respectively, to accord with the fabricated NR arrays as depicted in Fig. 4a. Other simulation parameters are similar to our previous report [29]. Field collected by monitor was used to integrate the power P_0_ that escape from the top surface, and the source power of dipole is defined as P_S_, so the LEE is *η* = *P*_0_/*P*_*S*_. And the extraction enhancement can be calculated by *E*_n_ = *η*_r_/*η*_p_, where *η*_p_, *η*_r_ is the LEE of PLA and NR samples, respectively. Figure 4 b shows the light extraction enhancement of the NR arrays compared with PLA sample at transverse electric (TE) and TM polarization states. One can see that for the n-type layer emission at approximately 265 nm, the LEE enhancement ratios are approximately 2.4 and 9.2 for the TE and TM polarization states, respectively. Previous investigation indicated that even for compressively grown AlGaN MQWs, strongly TE-polarized emission can be observed at wavelengths as short as 240 nm [30]. Thus, it is reasonable that the LEE enhancement of a mixture of TE and TM states is approximately 2.8. However, the LEE enhancement ratios are approximately 2.6 and 9.1 for the TE and TM polarization state, respectively, at approximately 277 nm. The calculated LEE enhancement ratio of the MQW emission from the experimental data is approximately 1.9, which is smaller than the simulated LEE enhancement ratio of both TE and TM polarization states. This may be attributed to the partly irregular shape of the experimentally fabricated NR arrays shown in Fig. 2a or the re-absorption of the damaged layer caused by the NIL process.

On the other hand, the reduced QCSE can enhance the IQE for the NR sample for the MQW emission. The IQEs of the n-type layer emission at 300 K can be estimated as approximately 1.96% [*I*_1_(PLA300K)/*I*_1_(PLA10K)] and 2.03% [*I*_1_(NR300K)/*I*_1_(NR10K)] for the PLA and NR samples, respectively. They are very close to each other because the QCSE does not exist in the n-type layer. However, the IQEs of the MQW emission at 300 K are approximately 1.32% [*I*_2_(PLA300K)/*I*_2_(PLA10K)] and 16.1% [*I*_2_(NR300K)/*I*_2_(NR10K)] for the PLA and NR samples, respectively. Thus the enhancement ratio of IQE is 12.2 for the MQW emission of the NR sample compared with the PLA sample. This great enhancement of the relative IQE should be attributed to the reduced QCSE of the NR sample. According to some similar works in blue/green LEDs [27, 31], a large strain relaxation due to the NR fabrication will reduce the QCSE effect. The reduced QCSE will increase the wave function overlap of electrons and holes and results in an increased IQE.

**Fig. 5:**
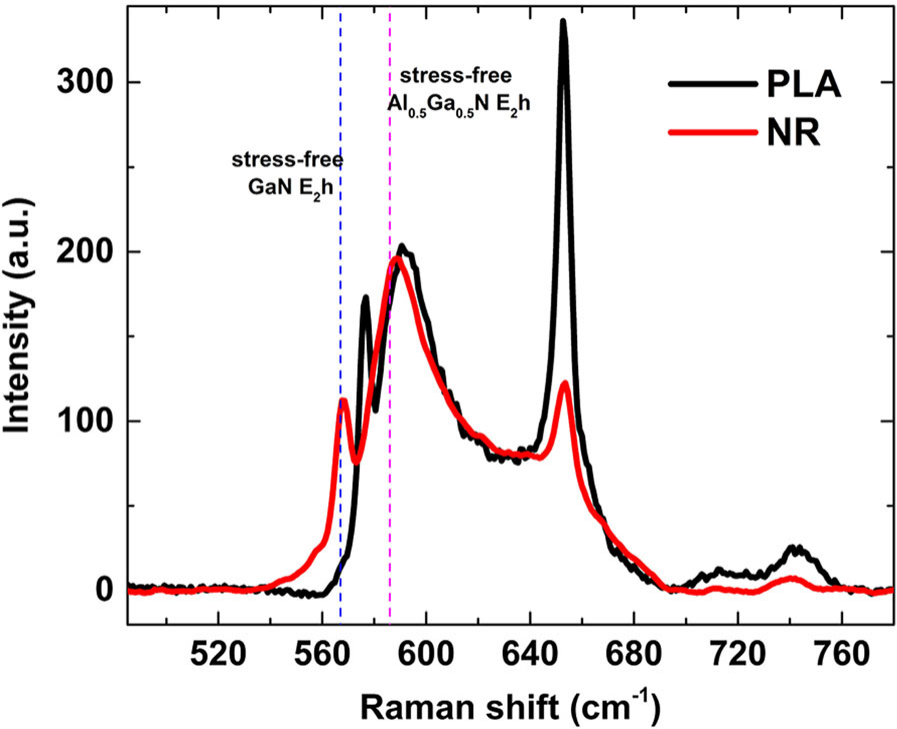
Raman spectra of the PLA and NR samples stimulated by a 514 laser at RT. The black and red curves represent PLA and NR samples, respectively. The blue and pink dashed lines correspond to *E*_2_h peak of unstressed GaN and Al_0.5_Ga_0.5_N, respectively

Raman measurement was performed to confirm the strain relaxation in the NR samples. Figure 5 shows the Raman spectra of the PLA and NR samples. The *E*_2_(high) phonon mode is usually utilized to characterize the stress state in the epitaxial layers. Notably, three *E*_2_(high) phonon modes are shown in the Raman spectra for both PLA and NR samples, corresponding to the GaN contact layer, n-type layer, and AlN buffer layer. Clearly, the peak shifts of PLA and NR samples are different compared with stress-free *E*_2_(high) phonon modes, indicating that the stress state has changed after the PLA sample was fabricated into the NR sample. Usually, the in-plane stress of the epitaxial layers is expressed by the following equation [29]:

1 $$ {\omega}_{{\mathrm{E}}_2\left(\mathrm{high}\right)}-{\omega}_0= C\sigma, $$

where C is the stress-shift rate (− 3.4 cm^−1^/GPa, − 3.1 cm^−1^/GPa, and − 3.25 cm^−1^/GPa for GaN, AlN, and Al_0.5_Ga_0.5_N, respectively) [29]. ${\omega}_{{\mathrm{E}}_2\left(\mathrm{high}\right)}$ and *ω*_0_ are the Raman shifts for the *E*_2_(high) mode of the corresponding epitaxial layers in our study and the stress-free materials, respectively. The *ω*_0_ values for GaN and Al_0.5_Ga_0.5_N are reported to be 567.0 and 586.0 cm^−1^ at RT, respectively [32]. The strain of the epitaxial layers can be expressed as [33]:

2 $$ {\sigma}_{\mathrm{xx}}=\left[{C}_{11}+{C}_{12}-2\frac{C_{13}^2}{C_{33}}\right]{\varepsilon}_{\mathrm{xx}}, $$

where *σ*_xx_ is the in-plane stress; *ε*_xx_ is the in-plane strain, and *C*_ij_ is the elastic constants of GaN and AlN given in previous report [34], i.e., a proportionality factor of 478.5 GPa for GaN, and 474.5 GPa for Al_0.5_Ga_0.5_N.

Using Eqs. (1, 2), the Raman shift, stress, and the strain are listed in Table 2. Notably, the strain is greatly reduced in the GaN contact layer. By simply considering a linear interpolation of the strain and stress in the different Al content epilayers, the stress/strain in the MQWs with 35% Al content can be obtained as 1.99 GPa/0.42% and 0.59 GPa/0.13% for the PLA and NR sample, respectively. Thus, a 69% strain has been relaxed in the MQWs layer of the NR sample.

**Table 2:** The *E*_2_(high) phonon frequencies (cm^−1^) observed at 300 K and the calculated stress and strain

	GaN			Al_0.5_Ga_0.5_N		
	Raman shift (cm^−1^)	Stress (GPa)	Strain	Raman shift (cm^−1^)	Stress (GPa)	Strain
PLA	576.6	− 2.82	− 0.59%	591.3	− 1.63	− 0.34%
NR	568.1	− 0.32	− 0.07%	588.3	− 0.71	− 0.15%

According to previous investigation [35], the polarization field *E*_w_ in the quantum wells can be expressed as

3 $$ {E}_{\mathrm{w}}=\frac{l_{\mathrm{b}}\left({P}_{\mathrm{b}}-{P}_{\mathrm{w}}\right)}{l_{\mathrm{w}}{\upvarepsilon}_{\mathrm{b}}+{l}_{\mathrm{b}}{\upvarepsilon}_{\mathrm{w}}}, $$

where *l*_w_, *l*_b_, *P*_w_, *P*_b_, and *ε*_b_, *ε*_w_ are the widths, total polarizations and dielectric constants of the wells and the barriers, respectively. Thus, not only the piezoelectric polarization but also the spontaneous polarization should be taken into account. The piezoelectric polarization is calculated by ${P}_{\mathrm{pz}}=2\left({e}_{31}-{e}_{33}\frac{C_{13}}{C_{33}}\right){\varepsilon}_{\mathrm{xx}}$ [36], where *e*_31_, *e*_33_, *C*_31_, and *C*_33_ are obtained by the linear interpolation from the related parameters of GaN and AlN [37, 38], the strain *ε*_xx_ is calculated by the Raman spectra using linear interpolation method. The spontaneous polarization is obtained by the linear interpolation from the spontaneous polarization of GaN and AlN [37, 39]. Thus, by using the dielectric constant of the wells and the barriers obtained by the linear interpolation from the dielectric constant of GaN *ε*_GaN_ = 8.9 and AlN *ε*_AlN_ = 8.5 [40], the polarization field can be calculated by Eq. (3). Table 3 lists the spontaneous polarization, piezoelectric polarization, total polarization, and polarization field in the quantum wells for PLA and NR samples; one can clearly see that the polarization field is reduced after the NR fabrication.

**Table 3:** Spontaneous polarization, piezoelectric polarization, total polarization, and polarization field in the quantum wells

	Spontaneous polarization (C/m^2^)	Piezoelectric polarization (C/m^2^)	Total polarization (C/m^2^)	Polarization field (C/m^2^)
PLA	− 0.0536	0.0066	− 0.047	− 0.00397
NR	− 0.0536	0.002	− 0.0516	− 0.00335

## Conclusion

In summary, highly uniform AlGaN NR arrays with MQWs embedded have been successfully fabricated by NIL and top-down etching techniques. Two peaks corresponding to the emission from the n-type layer (at higher energy) and MQWs (at lower energy) are observed by the CL measurement for both NR and PLA samples at 300 K and 10 K. For the n-type layer emission, an over 2-fold LEE enhancement has been observed while the IQE is hardly enhanced via the NR fabrication. For MQW emission, the LEE enhancement ratio can be estimated around 1.9 and a 12.2-fold IQE enhancement is achieved. Raman spectra demonstrated that the strain is reduced from 0.42% to 0.13% by the NR fabrication, showing a strong evidence of reduced QCSE. Our results indicated that for the samples without great crystal quality, the spatial separation between the electrons and the holes caused by the QCSE would be an important factor for the IQE reduction.

## Abbreviations

CL: Cathodoluminescence
EBL: Electron beam lithography
FDTD: Finite-difference time-domain
ICP: Inductively coupled plasma
LEE: Light extraction efficiency
LT: Low temperature
MOCVD: Metal-organic chemical vapor deposition
MQWs: Multi-quantum wells
Ni: Nickel
NIL: Nanoimprint lithography
NR: Nanorod
PECVD: Plasma-enhanced chemical vapor deposition
PLA: Planar
PVD: Physical vapor deposition
QCSE: Quantum-confined stark effect
RIE: Reactive-ion etching
RT: Room temperature
SEM: Scanning electron microscopy
TE: Transverse electric
TEM: Transmission electron microscopy
TM: Transverse magnetic
UV: Ultraviolet
